# Metagenomic characterization of the cecal microbiota community and functions in finishing pigs fed fermented *Boehmeria nivea*

**DOI:** 10.3389/fvets.2023.1253778

**Published:** 2023-09-28

**Authors:** Xiaoxiao Liang, Zhenya Zhai, Fengyun Ren, Yucheng Jie, Soo-Ki Kim, Kai-Min Niu, Xin Wu

**Affiliations:** ^1^College of Animal Science and Technology, Henan Agricultural University, Zhengzhou, China; ^2^Jiangxi Functional Feed Additive Engineering Laboratory, Institute of Biological Resource, Jiangxi Academy of Sciences, Nanchang, China; ^3^CAS Key Laboratory of Agro-Ecological Processes in Subtropical Region, Institute of Subtropical Agriculture, Chinese Academy of Sciences, National Engineering Laboratory for Pollution Control and Waste Utilization in Livestock and Poultry Production, Changsha, China; ^4^College of Animal Science and Technology, Hunan Agricultural University, Changsha, China; ^5^Department of Animal Science and Technology, Konkuk University, Seoul, Republic of Korea; ^6^Tianjin Institute of Industrial Biotechnology, Chinese Academy of Sciences, Tianjin, China

**Keywords:** pigs, metagenomics, *Boehmeria nivea*, fermentation, cecal microbiota

## Abstract

Ramie (*Boehmeria nivea*, BN) is used as livestock forage through suitable silage fermentation owing to its nutritional value. To date, relatively few studies have investigated the effects of dietary fermented BN (FBN) on gut health in finishing pigs. The aim of the present study was to investigate the effects of dietary supplementation with 20% FBN on intestinal morphology, gene expression, and the functional response of the gut microbiota in finishing pigs. We found that FBN did not significantly affect serum antioxidant enzyme activities, ileal morphology, or the expression of genes encoding antioxidant enzymes, inflammatory cytokines, or tight junction proteins in the liver of the pigs. However, the gene expression levels of aryl hydrocarbon receptor (*AHR*) and interleukin 6 (*IL6*) were significantly downregulated in the ileum. A metagenomic analysis demonstrated that, compared with that seen in the control group, the cecal microbiota of pigs in the FBN treatment group was more closely clustered and contained a greater number of unique microbes. Bacteria were the predominant kingdom in the cecal microbiota, while Firmicutes, Bacteroidetes, and Proteobacteria were the dominant phyla, and *Streptococcus*, *Lactobacillus*, and *Prevotella* were the dominant genera. Dietary FBN significantly increased the abundance of the probiotic bacterium *Roseburia inulinivorans* (*p* < 0.05). Functional analysis of the cecal microbiota showed that ABC transporter levels and glycolysis/gluconeogenesis-associated functions were diminished in FBN-fed pigs. Meanwhile, CAZyme analysis revealed that dietary FBN significantly downregulated the contents of carbohydrate-active enzymes, such as GT2, GH1, GH25, and GH13_31. In addition, cytochrome P450 analysis revealed that the abundance of CYP51 and CYP512 decreased with FBN treatment. An assessment of antibiotic resistance based on the Comprehensive Antibiotic Resistance Database (CARD) annotation indicated that the cecal microbes from pigs in the FBN treatment group had increased resistance to lincosamide, streptogramin, and chloramphenicol and reduced resistance to amikacin, isepamicin, neomycin, lividomycin, gentamicin, paromomycin, ribostamycin, and butirosin. Finally, virulence factor-related analysis showed that putative hemolysin-associated functions were decreased, whereas fibronectin-binding protein, flagella, and alginate-associated functions were increased. Taken together, our data showed that FBN supplementation exerted only minor effects on intestinal morphology and microbial community composition, suggesting that it is potentially safe for use as a supplement in the diets of finishing pigs. However, more studies are needed to validate its functionality.

## 1. Introduction

Over the past few decades, China has imported an enormous quantity of soybean meal (SBM) owing to the rapid scaling-up of the modern swine industry and the limited domestic supply of SBM. Researchers and feed manufacturers have increasingly focused on identifying cost-effective native alternatives to SBM to reduce the reliance on imported SBM and maintain the sustainable development of the Chinese swine industry. Ramie (*Boehmeria nivea*) is an ancient, fast-growing, perennial textile fiber crop widely cultivated in the south of China (1). The leaves and tender tops of ramie are low in fiber but high in protein (20% crude protein content on a dry matter basis) and rich in amino acids (especially lysine), minerals, flavonoids, polyphenols, and vitamins (1). Ramie has been applied in feed for black goats as a substitute for alfalfa (2) and has not impacted the health, meat quality, or gut microbiota diversity of Boer goats, even at an inclusion rate as high as 40% (3). Dietary ramie and rice straw mixed silage has been proven to increase milk fat percentage, milk solid content, and dry matter digestibility in dairy cows (4). Ramie has good palatability and nutritional value as a supplement in ruminant feed but remains underutilized. Ramie has also been used in the diets of finishing pigs to improve carcass traits, muscle chemical composition (5), serum antioxidant enzyme activities, and pork fatty acid composition (6). A low dietary inclusion level of ramie has been reported to increase the average daily gain and feed-to-gain ratio in pigs, although higher inclusion levels adversely affected the growth performance of the animals (6).

Silage is one of the best methods for preserving forage resources in regions with heavy rainfall because it contains high levels of lactic acid bacteria and organic acids and low levels of non-digestible fiber (7). In general, silage is produced through natural bacteria-mediated fermentation, with some additives being included to improve silage quality. *Aspergillus niger* is commonly used as an inoculant to treat a wide range of agricultural and household waste residues for the production of value-added products owing to its multi-enzymatic (mannanase, xylanase, cellulase, glucanase, and lipase) activities (8, 9). *A. niger* is also used for the production of organic acids and the hydrolysis of bound polyphenols using low-value materials (10, 11).

The gut microbiota and feed ingredients can mutually affect the host’s nutrition, metabolism, and overall health (12–14). However, it is not possible to predict how the introduction of a new feed ingredient into the diet of pigs will affect the development of the gut microbiota of the animals. The advancements in high-throughput sequencing technology have enabled a more comprehensive analysis of complex gut microbiota (15). Studies have traditionally employed 16S rRNA gene sequencing of fecal samples to analyze the gut microbiota diversity and composition (16); nevertheless, this method cannot achieve a thorough understanding of the effects of diet on the functionality of gut microorganisms. In this study, we prepared ramie silage using an autochthonous *A. niger* strain previously isolated from ramie and explored its effects on finishing pigs. We performed a metagenomic shotgun sequencing analysis of the cecal content of the animals to comprehensively elucidate how a high inclusion level of ramie silage affects their gut microbiota. The effects of dietary ramie silage on ileal morphology and expression of antioxidant and anti-inflammatory genes were also evaluated.

## 2. Materials and methods

### 2.1. Animals and experimental diets

All experimental procedures involving animals were approved (2015-8A) by the Animal Care and Use Committee of the Institute of Subtropical Agriculture, Chinese Academy of Sciences. A total of 12 DLY (Duroc × Landrace × Yorkshire) finishing pigs with an average initial body weight of 60.11 ± 0.09 kg were used in the study. A basal diet containing corn, SBM, and rice bran was formulated based on the recommendations of the National Research Council (Supplementary Table S1). The pigs were randomly allocated to two treatment groups, with six replicates per group. Pigs in the control (Con) group were fed a basal diet, and those in the fermented *Boehmeria nivea* (FBN) group were fed a basal diet in which the corn, SBM, and rice bran mixture were replaced by the isocaloric and isonitrogenous inclusion of 20% FBN. The fermented ramie was provided by Hunan Agricultural University. The ramie silage was prepared via the inoculation of 100 mL of 1 × 10^6^ CFU/mL *A. niger* into 1 kg of ramie powder. After packaging and fermenting at 30°C for 60 days, the FBN was formulated and pelleted. The pigs were provided with feed twice a day and had free access to their diets. The feeding trial lasted for 60 days after a 7 day adaptation period.

### 2.2. Euthanization and sample collection

At the end of the feeding experiment, the pigs were euthanized according to standard commercial procedures. Samples of blood, liver, ileum, and colon contents were collected from each pig. The blood was collected from the jugular vein as previously described (17). Serum samples were prepared by centrifugation of the blood at 3,000 × *g* for 10 min at 4°C and stored at −20°C for analysis of antioxidant enzyme activity. Liver and ileal tissue samples and cecal contents (squeezed out from the cecum) were snap-frozen in liquid nitrogen and stored at −80°C for gene expression analysis and metagenomic shotgun sequencing.

### 2.3. Serum antioxidant parameters

Serum total antioxidant capacity (TAC) and superoxide dismutase (SOD), catalase (CAT), and glutathione peroxidase (GSH-Px) activities were determined using commercially available kits obtained from Suzhou Comin Biotechnology Co., Ltd. (Suzhou, Jiangsu, China) following the manufacturer’s instructions.

### 2.4. Histological analysis of ileal morphology

Paraffin-embedded ileal tissue sections (5 μm) were stained with hematoxylin and eosin (H&E). Ileal tissue integrity was evaluated by measuring the villus length (VL) and crypt depth (CD) and determining the VL/CD ratio under a light microscope (Olympus, Japan) fitted with a digital camera.

### 2.5. Quantitative real-time PCR (qPCR) analysis of gene expression in the ileum and liver

Total RNA extraction, cDNA synthesis, and qPCR were performed using previously described methods (5). Briefly, total RNA was extracted from 0.1 g of liver or ileal tissue using column RNA extraction kits (Magen, Guangzhou, China). RNA concentration and purity were determined using a NanoDrop 2000 spectrophotometer (Thermo Fisher Scientific, Waltham, MA, USA) and 1% agarose gel electrophoresis, respectively. Approximately 1 μg of the isolated RNA was reverse transcribed into cDNA using a cDNA synthesis kit (CWBIO, Jiangsu, China) following the manufacturer’s instructions. qPCR was conducted on an ABI 7900HT Real-Time PCR System (Applied Biosystems, Branchburg, NJ, USA). The primers used in the study were designed with Primer3 and BLAST using default parameters (Premier Biosoft International, Palo Alto, CA, USA) and are described in Supplementary Table S2. The PCR cycling conditions were as follows: 95°C for 10 min, followed by 40 cycles of 95°C for 15 s and 60°C for 60 s, and then 1 cycle of 72°C for 30 s. Relative gene expression levels were calculated using the 2^–ΔΔCt^ method after normalization to *GAPDH* or β-actin (18).

### 2.6. Microbial DNA extraction and metagenomic sequencing

Genomic DNA was extracted from cecal content using an E.Z.N.A. Soil DNA Kit (Omega Bio-Tek, Norcross, GA, USA) according to the manufacturer’s protocol. DNA concentration and quality were assessed using a NanoDrop 2000 spectrophotometer (Thermo Fisher Scientific, Waltham, MA, USA) and 1% agarose gel electrophoresis, respectively. Metagenomic DNA was paired-end sequenced (400 bp fragments) on the NovaSeq/HiSeq X 10 platform (Illumina Inc., San Diego, CA, USA) at Majorbio Bio-Pharm Technology Co., Ltd. (Shanghai, China) using NovaSeq/HiSeq X 10 reagent kits.

### 2.7. Analysis of sequencing data

The sequencing data were analyzed online on the Majorbio Cloud Platform.^1^ Low-quality reads were removed using fastp^2^ (19). Reads were aligned using the Burrows-Wheeler Aligner (BWA)^3^ (20). Contigs of at least 300 bp were selected and used for further gene prediction and annotation. Open reading frames (ORFs) from each assembled contig were predicted using MetaGene^4^ (21). The predicted ORFs (length ≥ 100 bp) were retrieved and translated into amino acid sequences based on the NCBI translation table.^5^ A non-redundant gene catalog was constructed using CD-HIT^6^ (22) with 90% sequence identity and 90% coverage. After quality control, the reads were mapped to the non-redundant gene catalog with 95% identity using SOAPaligner^7^ (23), and the gene abundance in each sample was calculated.

### 2.8. Functional annotation

Representative sequences from the non-redundant gene catalog were aligned to the NCBI non-redundant database for taxonomic annotation and Cluster of Orthologous Groups (COG) analysis of proteins. Functional annotation of the representative sequences against the Kyoto Encyclopedia of Genes and Genomes (KEGG)^8^ was performed using Diamond^9^ (24). In addition, functional annotation of the representative genes was further analyzed in the Carbohydrate-Active enZYme (CAZy) database (v07312018)^10^, the Comprehensive Antibiotic Resistance Database (CARD)^11^, and the Virulence Factor Database (VFDB)^12^ using Diamond (24).

### 2.9. Statistical analysis

Differences between the Con and FBN treatments were assessed using the *t*-test in SPSS 24.0 (SPSS IBM, NY, USA). The results were expressed as mean ± standard error of the mean (SEM), and a *p*-value <0.05 was considered significant.

## 3. Results

### 3.1. Serum antioxidant parameters

The effects of dietary FBN on serum antioxidant enzyme activities in finishing pigs are shown in Table 1. Serum TAC, SOD, CAT, and GSH-Px activities were not significantly affected by the supplementation of 20% FBN in the diet.

**Table 1:** Dietary effect of fermented *Boechmeria nivea* leaves on serum antioxidant enzymes.

Items	Con	FBN	*p* value
TAC (U/mL)	1.82 ± 0.17	1.24 ± 0.10	0.055
SOD (U/mL)	38.09 ± 8.53	37.25 ± 7.01	0.992
CAT (nmol/min/mL)	22.90 ± 1.47	20.36 ± 0.62	0.287
GSH-Px (nmol/min/mL)	15.84 ± 3.84	16.44 ± 3.46	0.992

### 3.2. Ileal morphology

**Figure 1:**
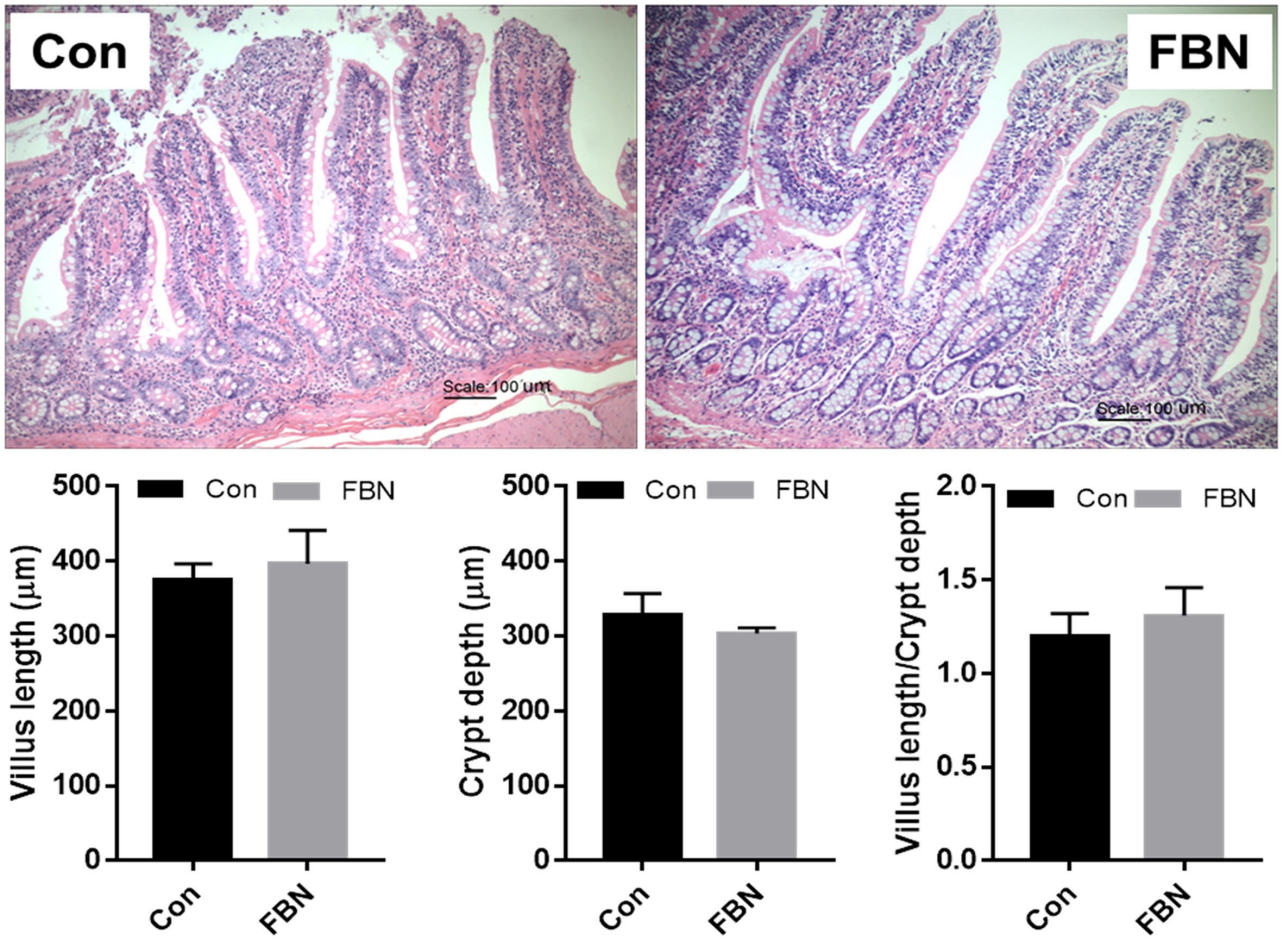
Dietary effect of fermented *Boechmeria nivea* on ileal morphology of finishing pigs.

The effects of dietary FBN on ileal histomorphology are shown in Figure 1. No significant differences in VL, CD, or VL/CD ratio were found between the two treatment groups (*p* > 0.05).

### 3.3. Gene expression in the liver and ileum

**Figure 2:**
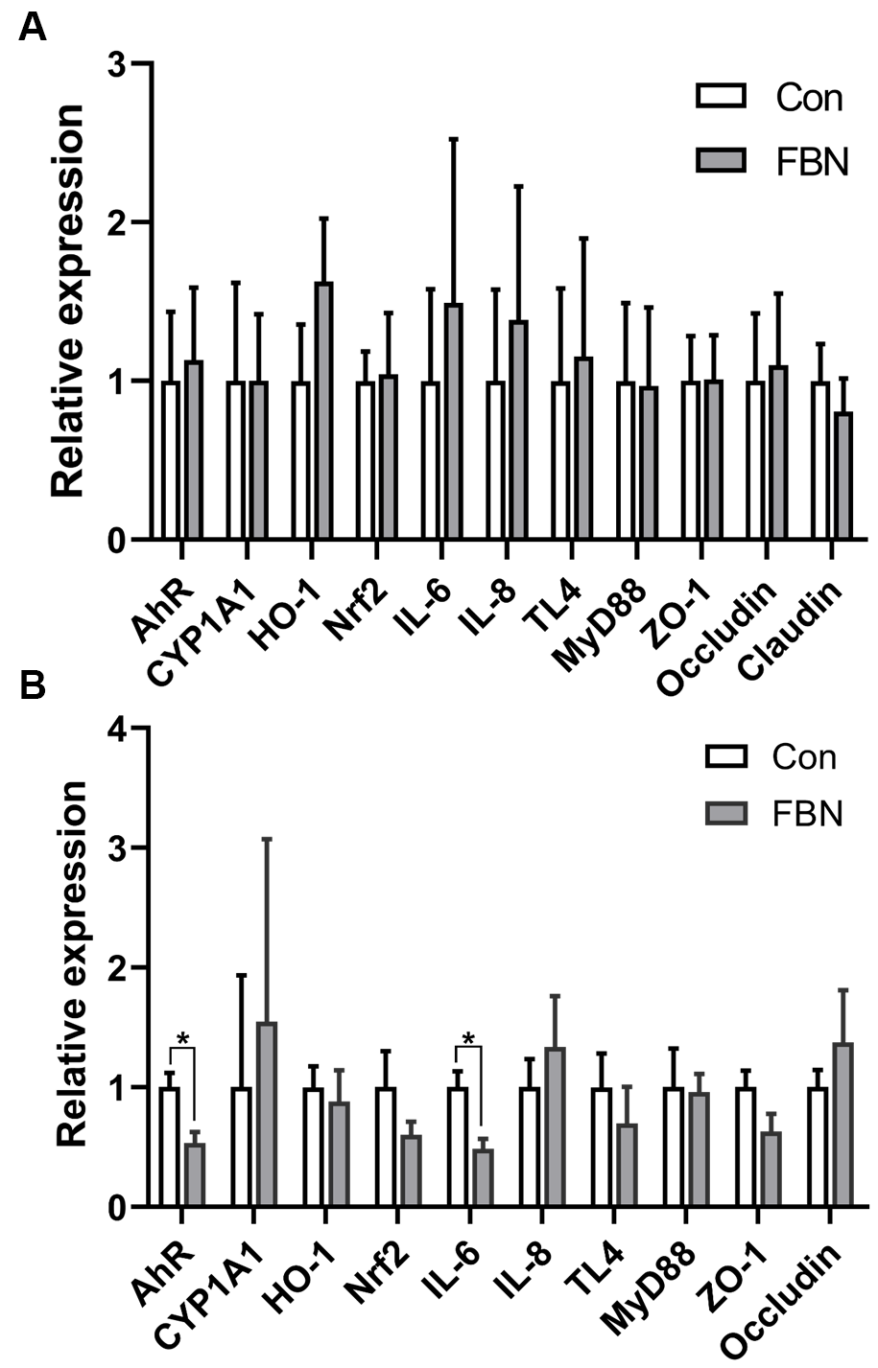
Dietary effects of fermented *Boechmeria nivea* on relative gene expression in liver **(A)** and ileum **(B)** of finishing pigs.

Compared with the Con treatment, no significant changes in the relative expression levels of genes encoding antioxidants, inflammatory cytokines, and tight junction proteins were detected in the livers of animals administered with FBN (Figure 2A); however, the expression of the *AHR* and *IL6* genes in the ileum was significantly downregulated by dietary FBN supplementation (*p* < 0.05) (Figure 2B).

### 3.4. Taxonomic composition of the cecal microbiomes of the two groups

**Figure 3:**
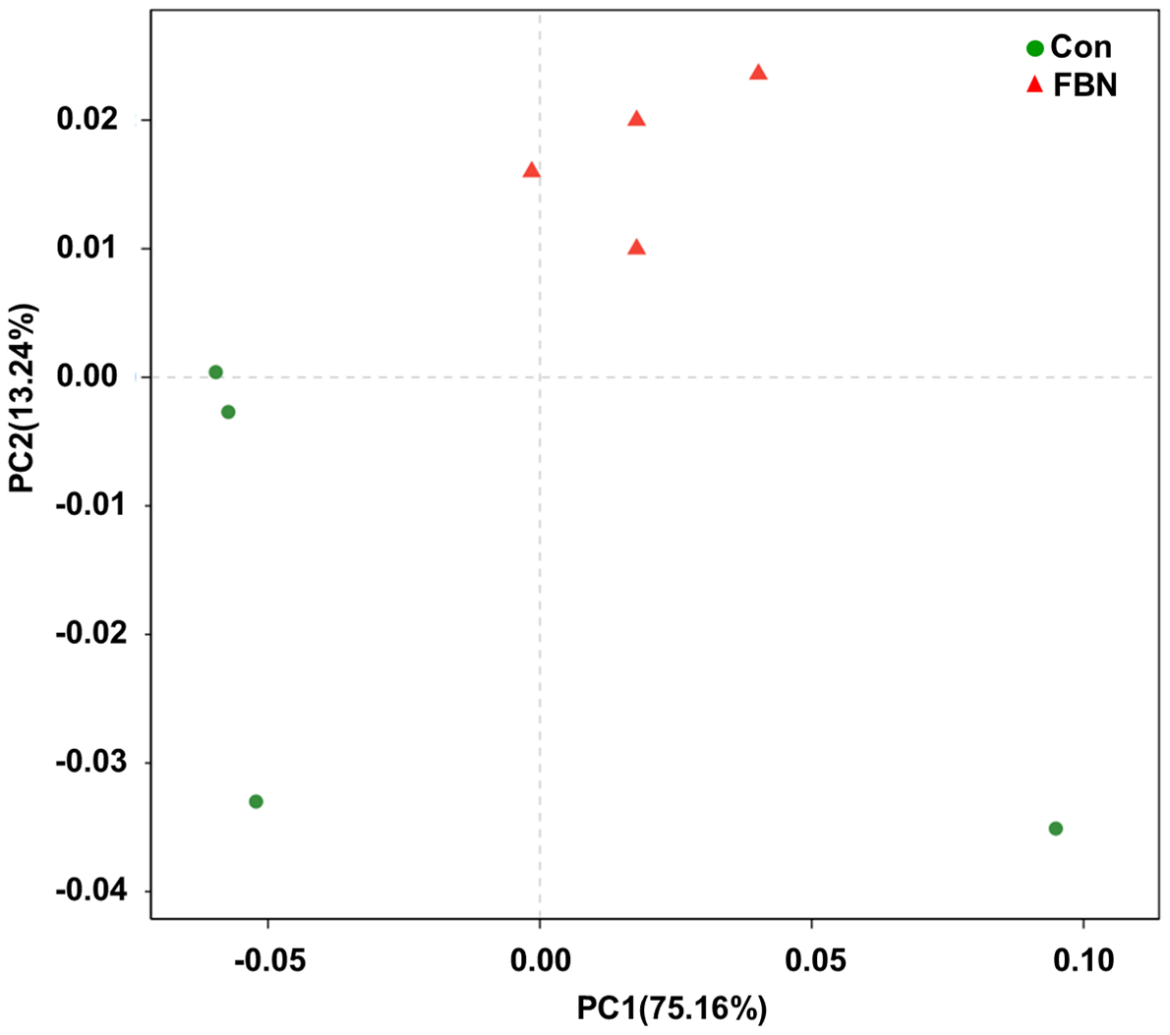
Principal component analysis of the similarity of the cecal microbiota.

**Figure 4:**
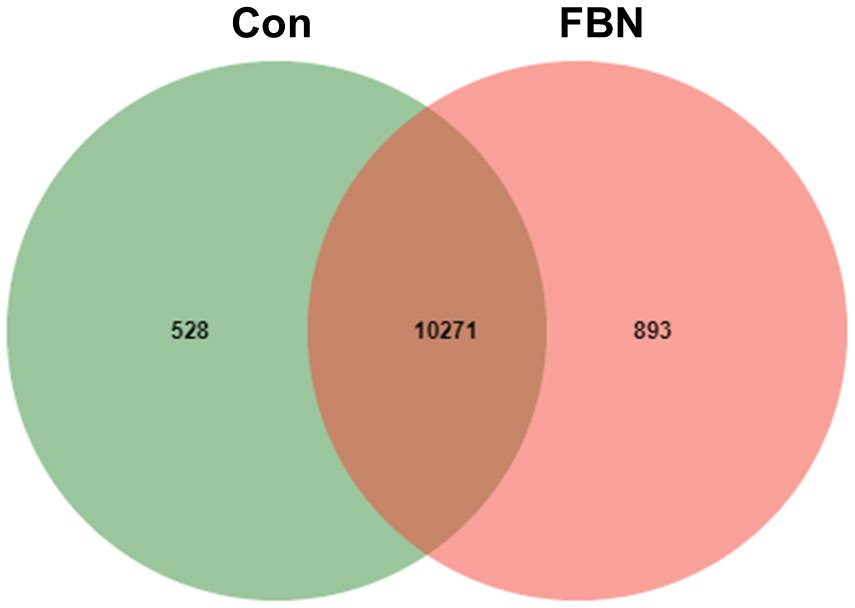
Venn diagram showing the unique and shared species in the cecal microbiota.

**Figure 5:**
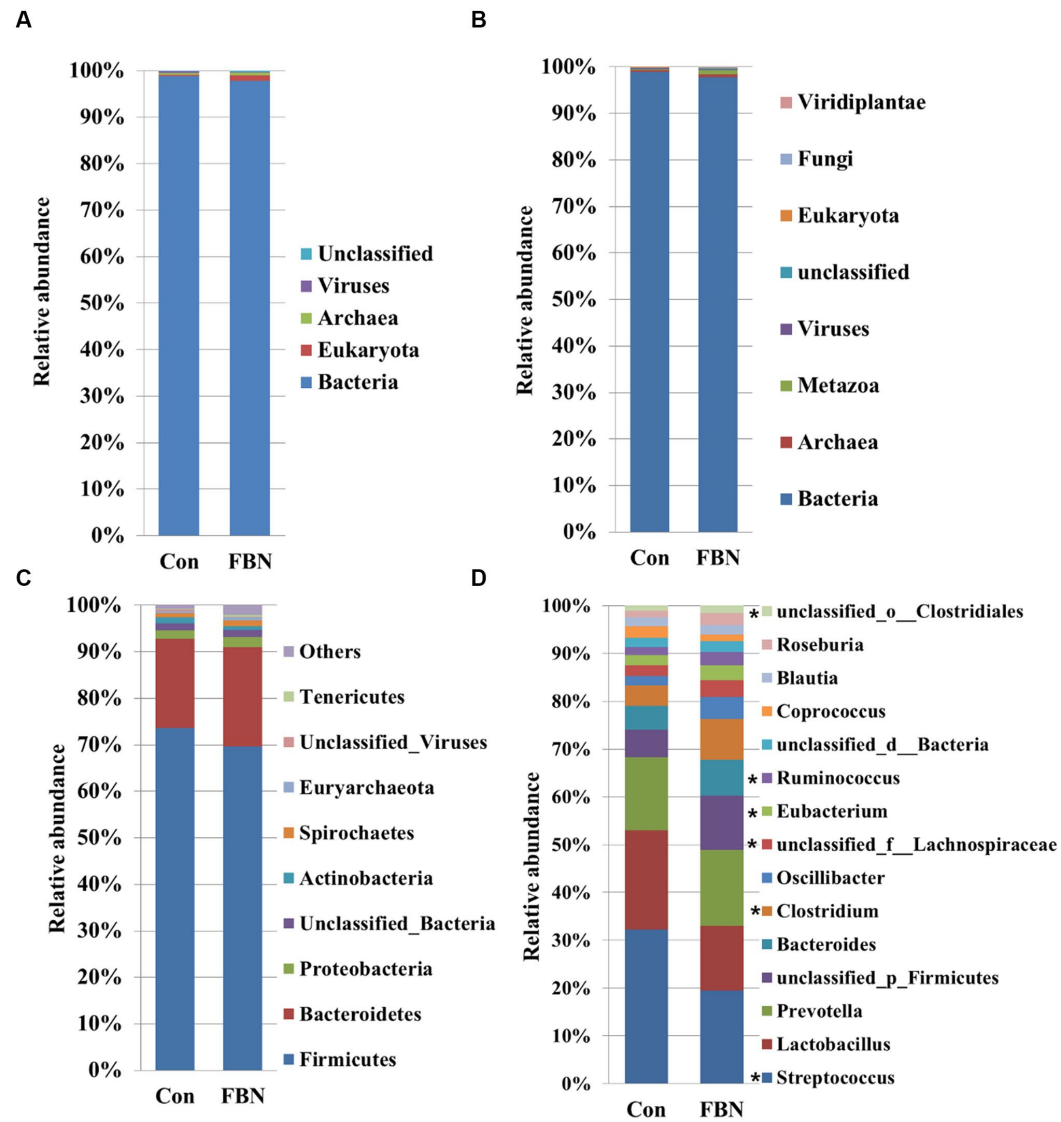
The dominant cecal microbiome at the domain **(A)**, kingdom **(B)**, phylum **(C)**, and genus **(D)** levels. *indicates significant differences at the level *P* < 0.05.

Principal component analysis (PCA) indicated that the cecal microorganisms were more closely clustered in the FBN treatment group than in the Con treatment group, and there was a distinct separation between the microbiota of the two groups (Figure 3). A total of 11,692 species were identified using shotgun metagenomic sequencing, of which 10,271 were shared between the two groups and 528 and 893 were unique to the Con and FBN groups, respectively (Figure 4). The taxonomic composition of the cecal microbiome was determined at the domain, kingdom, phylum, and genus levels (Figure 5). Bacteria were the most abundant organisms at the domain and kingdom levels, with other organisms making up only a very small proportion of the total (Figures 5A,B). At the phylum level, Firmicutes, Bacteroidetes, and Proteobacteria were predominant in that order of abundance (Figure 5C). At the genus level, 15 dominant genera were identified in the cecum of the finishing pigs. Dietary FBN supplementation significantly reduced the relative abundance of *Streptococcus* and *Ruminococcus*, and increased that of *Clostridium*, unclassified_f_*Lachnospiraceae*, *Eubacterium*, and unclassified_o_Clostridiales in the cecal contents of the animals (Figure 5D).

### 3.5. Functional annotation of the microbiome

**Figure 6:**
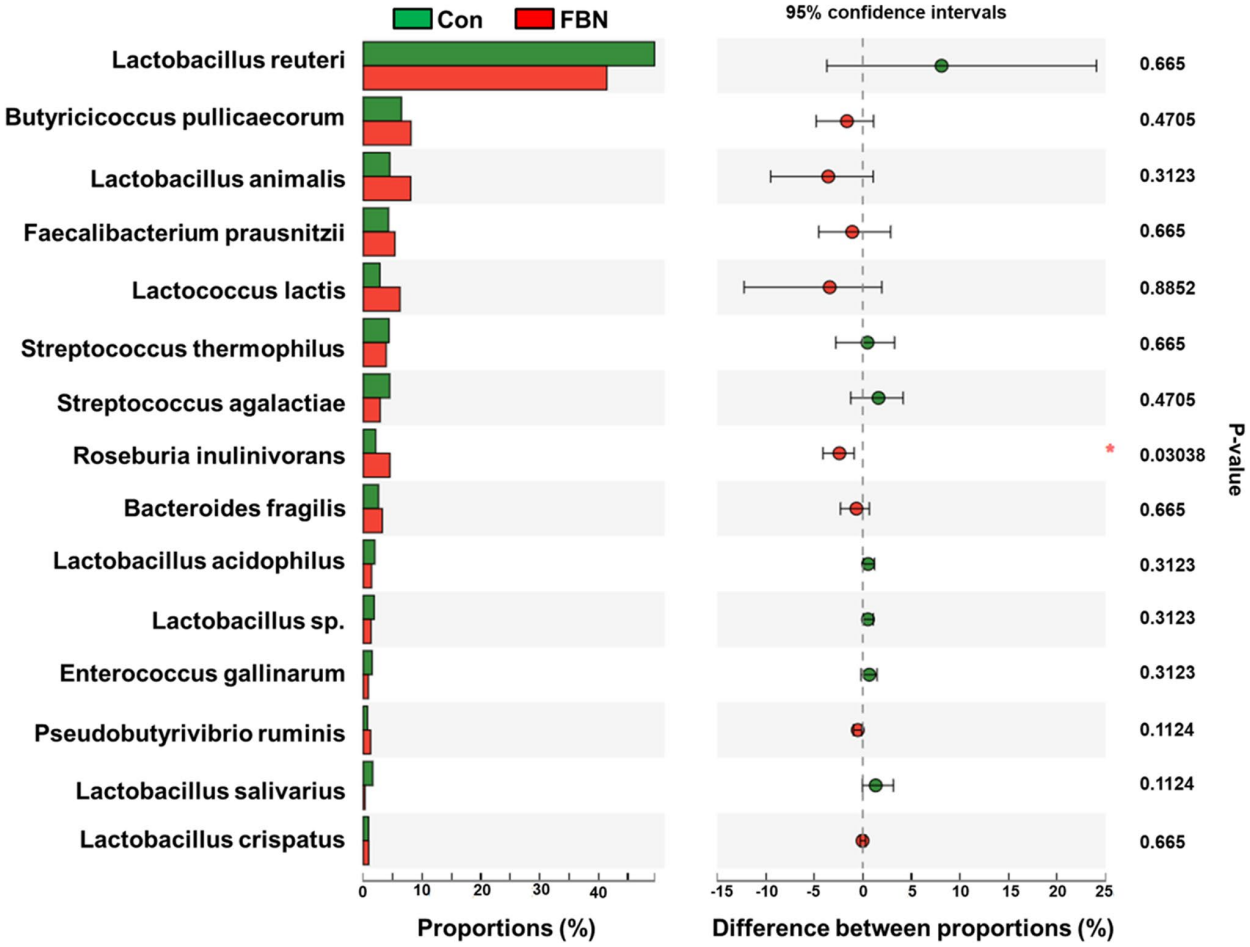
Putative probiotic abundance in the cecal microbiota annotated by the probiotic database.

**Figure 7:**
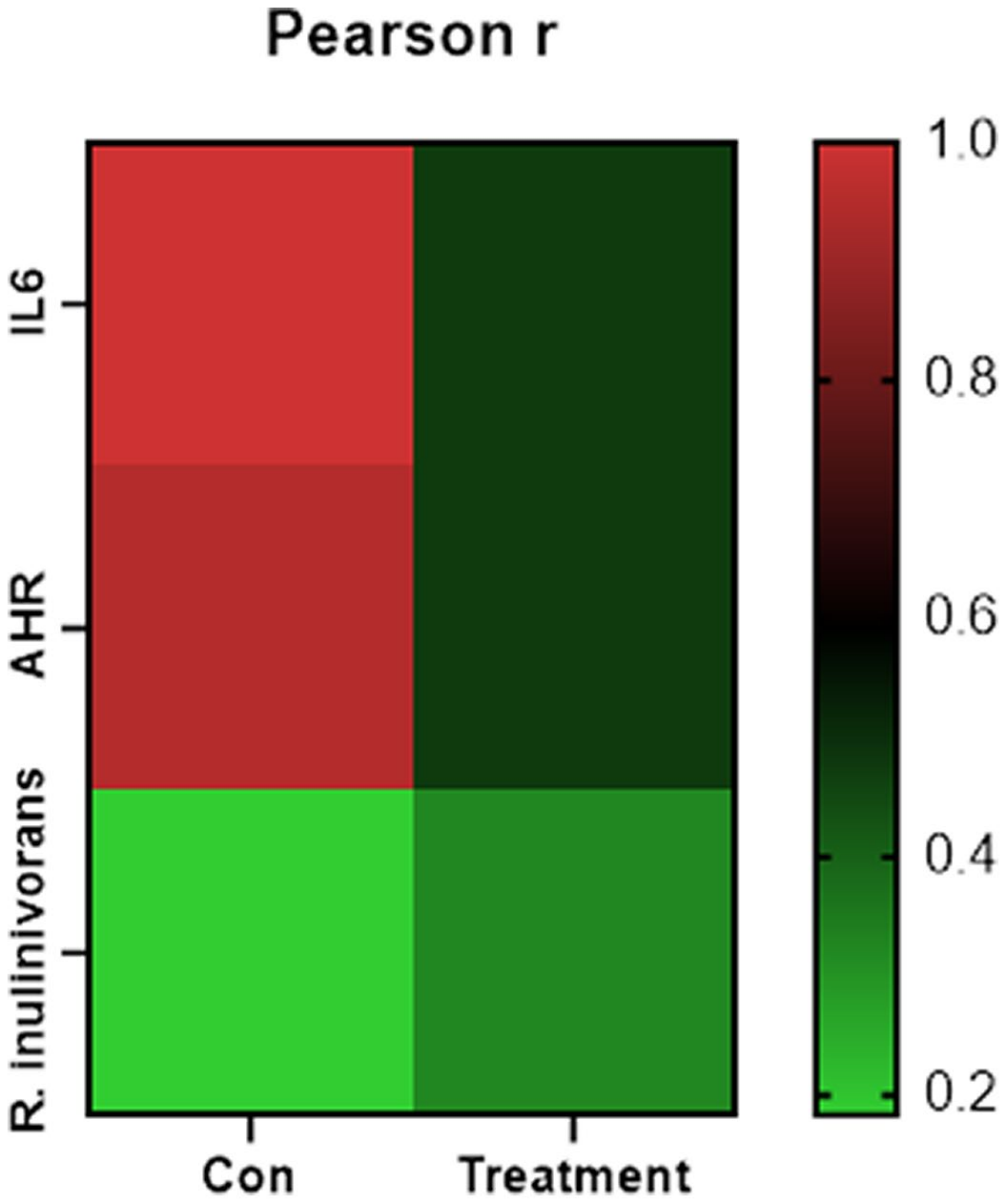
Correlation analysis between the differentially altered species and gene expression.

**Figure 8:**
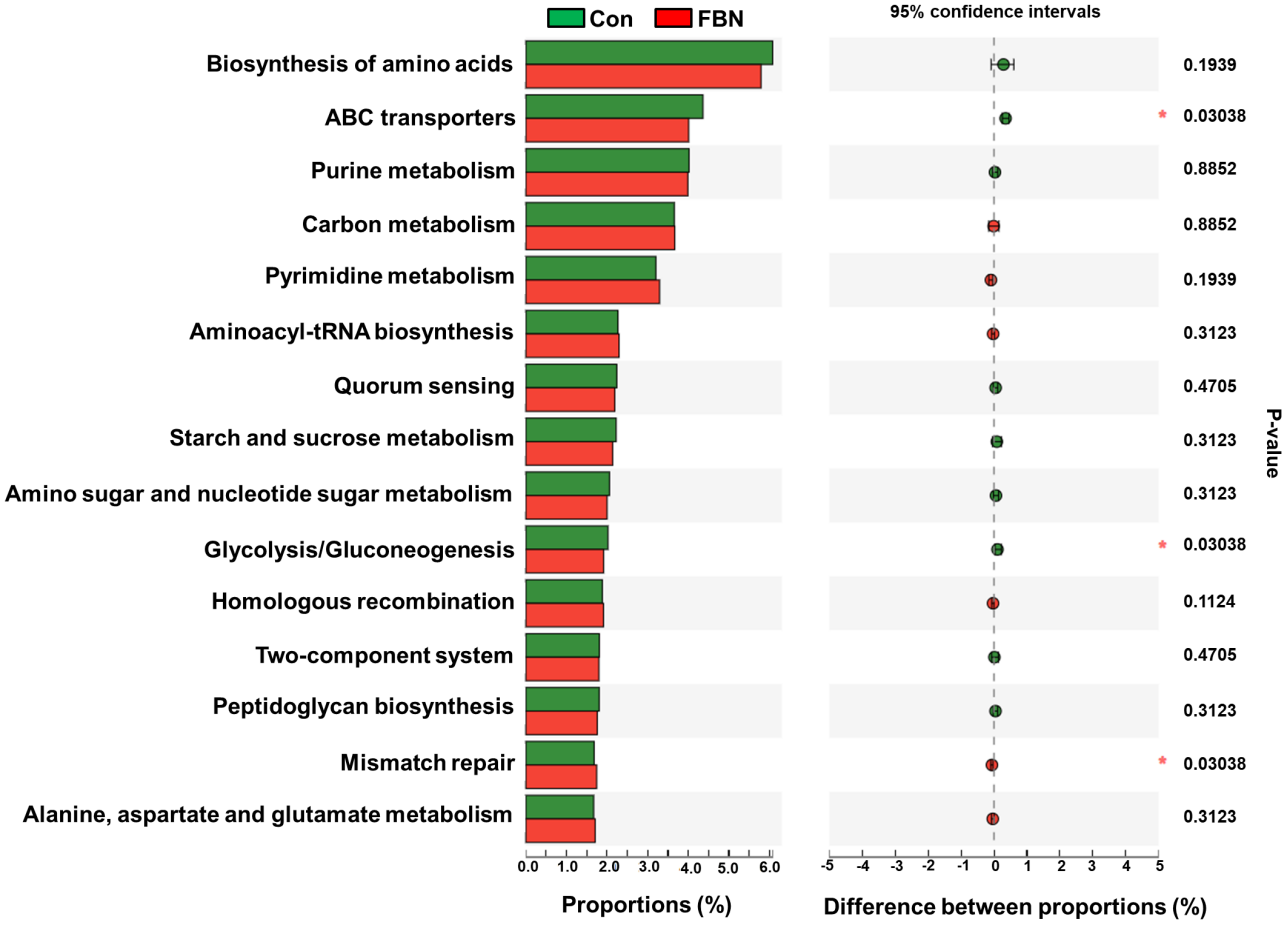
Putative functions of the cecal microbiome annotated by the Kyoto Encyclopedia of Genes and Genomes (KEGG) at level 3.

**Figure 9:**
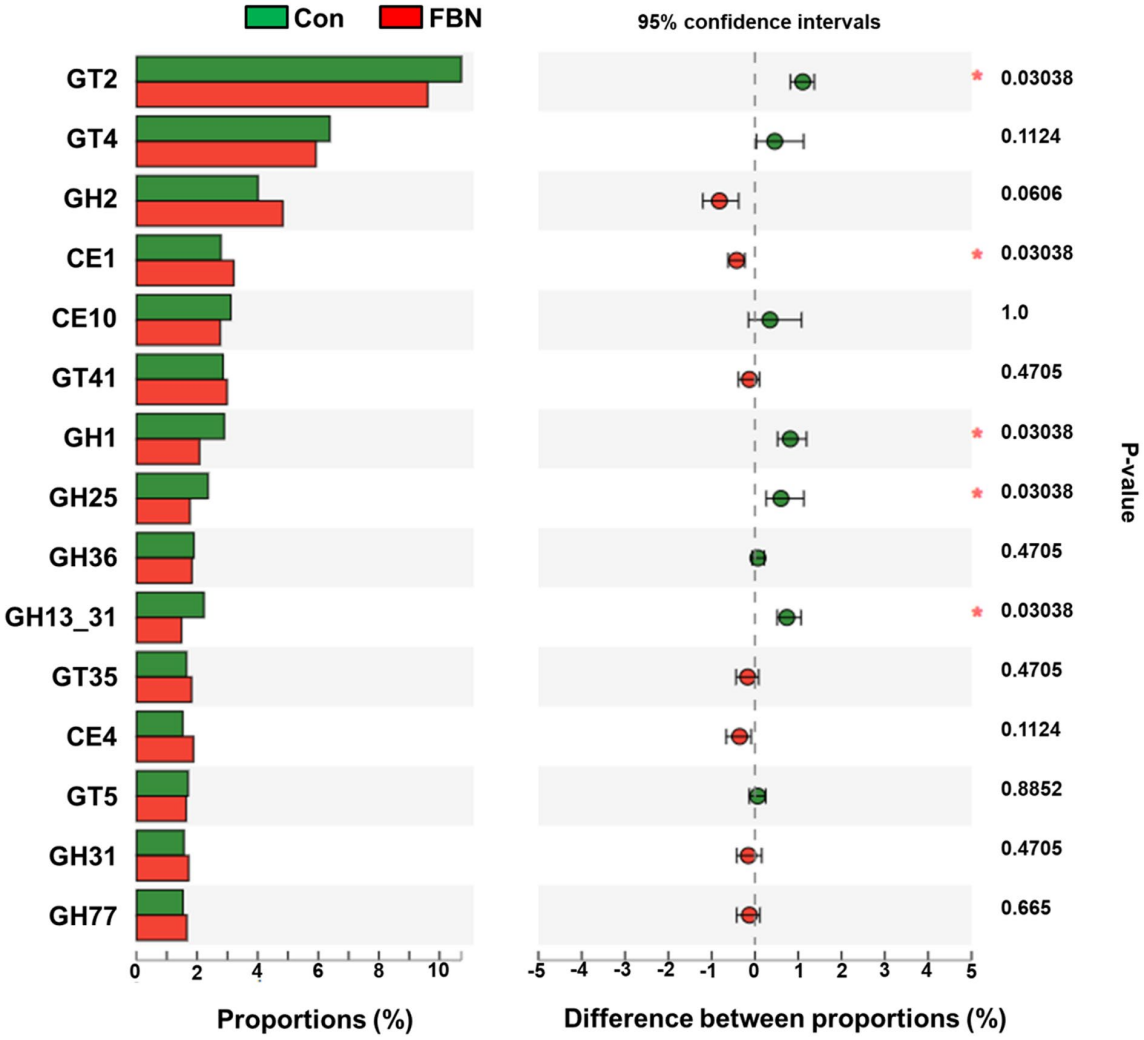
Putative carbohydrate-active enzyme (CAZyme) abundances of cecal microbes at the family level, annotated using the CAZy database. GT, glycosyltransferases; GH, glycoside hydrolases; CE, carbohydrate esterases.

**Figure 10:**
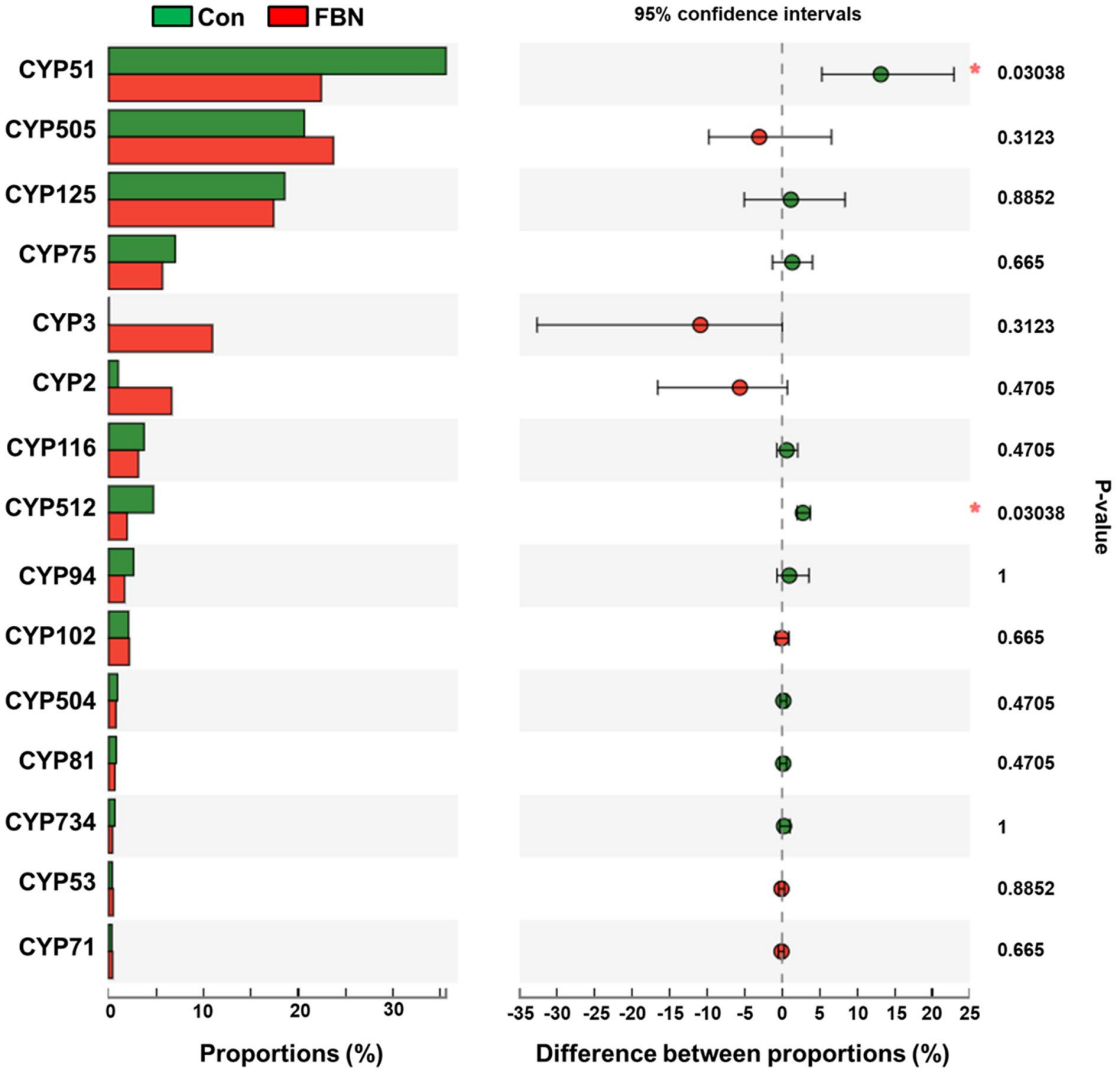
Putative CYP450 family of cecal microbes annotated using the Cytochrome P450 Engineering database.

**Figure 11:**
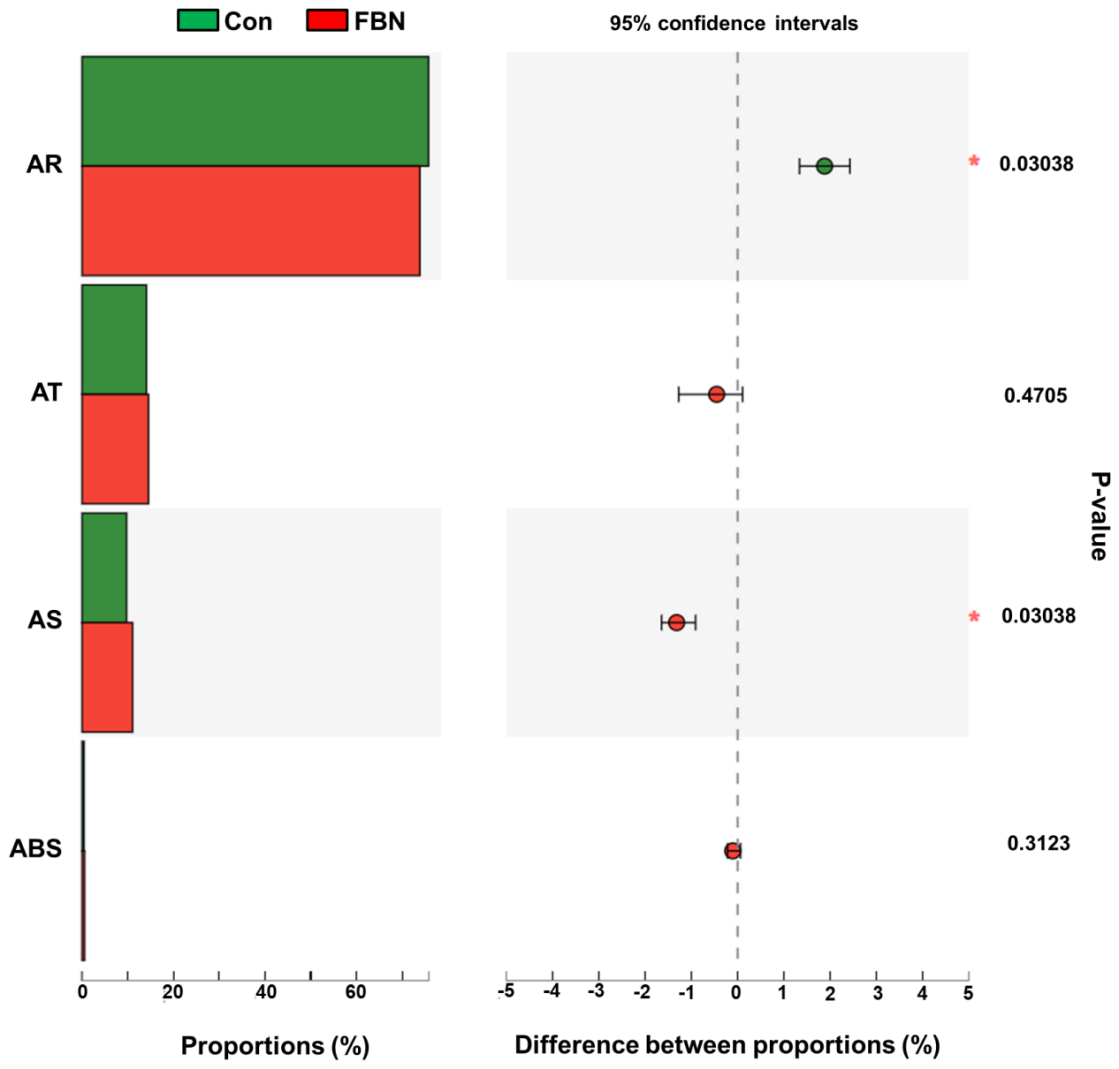
Putative antibiotic resistance of cecal microbes at the class level annotated using the CARD database (Comprehensive Antibiotic Resistance Database). AR, Antibiotic resistance; AT, Antibiotic Target; AS, Antibiotic Sensitive; ABS, Antibiotic Biosynthesis.

**Figure 12:**
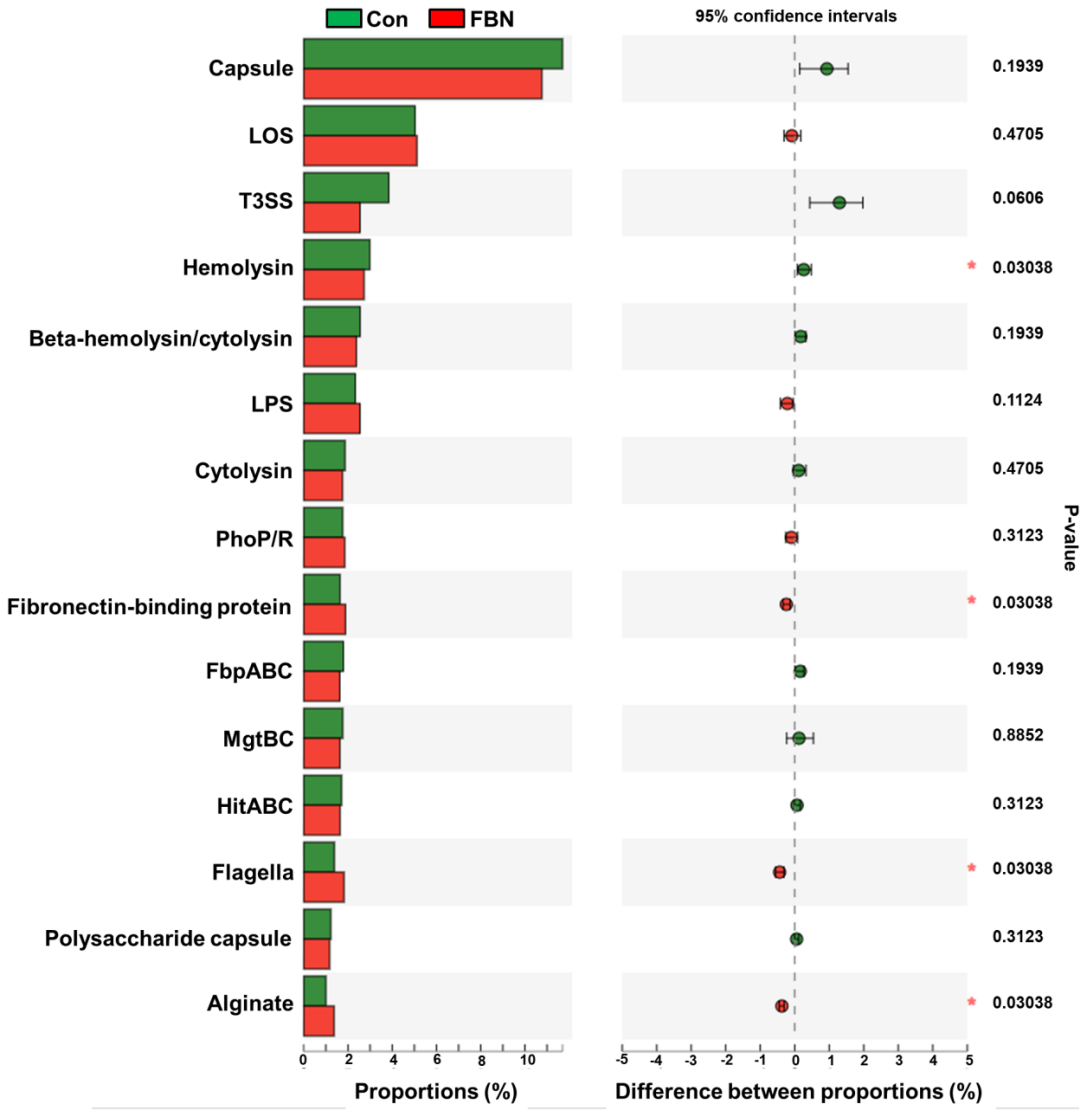
Putative virulence factors of cecal microbes annotated by the Virulence Factor Database (VFDB).

Changes in the functional profiles of the cecal microbial communities were investigated using Tax4FUN software based on the 16S rRNA gene datasets. To analyze the putative functionality of the microbiome, the genes were annotated based on six functional annotation databases. The sequences in the final gene catalog were aligned and analyzed in the probiotic database. A total of 15 dominant probiotic strains were identified among the sequences of the cecal microbiome of finishing pigs, with *Lactobacillus reuteri* exhibiting the greatest abundance. Additionally, the abundance of *Roseburia inulinivorans* was significantly improved by FBN treatment (Figure 6). The correlation analysis showed that the abundance of *R. inulinivorans* was negatively correlated with the gene expression of AHR and IL6 in the ileum (Figure 7). The gene catalog was then functionally annotated in the KEGG database, and the top 15 functional categories were identified (at level 3). Genes involved in pathways predicted to be related to ABC transporters and glycolysis/gluconeogenesis were significantly underrepresented, whereas those associated with mismatch repair were enriched with dietary FBN treatment (Figure 8). The gene catalogs were further aligned to their protein sequences and analyzed in the CAZy database. The sequences were mainly classified into three enzyme groups, namely, glycosyl transferases (GTs), glycoside hydrolases (GHs), and carbohydrate esterases (CEs). Among them, GT2, GH1, GH25, and GH13_31 were significantly reduced by dietary FBN treatment, coupled with a decline in CE1 abundance (Figure 9). Moreover, the gene sequences were analyzed in the Cytochrome P450 Engineering Database. The results showed that CYP51, CYP505, and CYP125 were the predominant CYP450 families. Among these P450 families, the abundance of CYP51 and CYP512 was significantly decreased with dietary FBN supplementation (Figure 10). We also analyzed the gene catalog in the CARD to identify and compare the abundance of antibiotic resistance genes (ARGs) (Figure 11). The results showed that dietary FBN reduced the abundance of ARGs and increased that of antibiotic susceptive genes. Finally, we analyzed the virulence factors in the cecal microbiome in the VFDB. We found that the abundance of hemolysin-related genes was decreased, whereas that of genes related to fibronectin-binding protein, flagella, and alginate was significantly increased in the FBN supplementation group (Figure 12).

## 4. Discussion

The use of ramie as an unconventional forage source in the diet of pigs has been explored in some areas of southern China with the aim of reducing the region’s reliance on imported SBM (5, 6). It has been reported that the inclusion of ramie powder at less than 9% in the diet of finishing pigs partially improved carcass traits and serum antioxidant capacity without impairing growth (5, 6). Similarly, in this study, we did not observe any detrimental effect of FBN on the activities of antioxidant enzymes in serum when supplemented at 20% in the diets of finishing pigs. Antioxidant enzymes such as SOD, CAT, and GSH-Px play important roles in the elimination of ROS and the maintenance of redox homeostasis (6). The fact that serum antioxidant enzyme contents were unaltered suggests that even high inclusion levels of FBN do not induce oxidative stress in finishing pigs.

In goats, high dietary inclusion levels of ramie (up to 40%) have been reported to improve butyrate concentrations in the rumen without altering the diversity of the ruminal microbiota (3). Compared with monogastric animals, ruminants are better at digesting ramie forage. During silage fermentation, the fiber content is typically reduced, which enhances the digestibility of the resulting forage. This implies that even a high inclusion level of fermented ramie may not be detrimental to pigs. Nevertheless, when a new ingredient is introduced into animal feed, the effects on gut health must be carefully assessed. In this study, no changes in intestinal architecture or morphology were observed in the FBN treatment group, indicating that FBN can be safely included in the diets of finishing pigs. Given its detoxifying functions, it is also important to assess the status of the liver when a new feed ingredient is introduced. AhR has been extensively studied for its role in mediating xenobiotic metabolism; however, it is also known to be a critical regulator of immunity and inflammation (25, 26). One study reported that a microbial polyphenol metabolite enhanced gut barrier function via activation of AhR/nuclear factor erythroid 2-related factor 2 (Nrf2)-dependent pathways (27). Nrf2 is a downstream target of the AhR and plays a vital role in the antioxidant defense system (28). We assessed the expression of genes involved in the AhR-Nrf2 pathway, such as those involved in antioxidant, immune, and barrier functions, in both the liver and the ileum. We found that the expression of both the *AHR* and *IL6* genes was downregulated in the ileum, which may be due to the presence of unknown phytochemicals in the FBN. IL-6 is a pro-inflammatory cytokine and is also involved in immune responses (29). The observed decline in *IL6* gene expression suggests that FBN has anti-inflammatory potential.

The gut microbiota plays a critical role in nutrient digestion and adsorption, gastrointestinal immunity, and epithelial cell renewal (30). Many factors, both intrinsic and extrinsic, can influence gut microbial diversity, composition, function, and metabolic activities. To date, no study has investigated the effects of dietary FBN on the gut microbiota of finishing pigs. Knowledge of the alterations that occur in the composition and relevant functions of the gut microflora in response to dietary changes will help in the development of practical new forage for pigs. In this study, FBN treatment-induced changes in the composition of the cecal microbiota were characterized by a greater number of unique species compared to the control treatment. Dietary FBN did not significantly affect microbial composition at the domain, kingdom, and phylum levels, suggesting that even high dietary inclusion levels of FBN do not alter gut microbial stability. At the genus level, the abundance of *Streptococcus* was reduced, while that of *Clostridium*, Unclassified_f_*Lachnospiraceae*, *Eubacterium*, *Ruminococcus*, Unclassified_o_*Clostridiales*, and the probiotic *Roseburia inulinivorans* was increased when FBN was included in the diet of the pigs. *Streptococcus*, *Lactobacillus*, and *Clostridium* species are prevalent in specific pig species, as revealed by a meta-analysis of 16S rRNA sequences and metagenomics (31). *Streptococcus* species such as *S. alactolyticus* are prevalent in pigs from China but not in pigs from France or Denmark, which may be attributed to the widespread use of antibiotics in China (31). Several ARGs that confer resistance to aminoglycosides, macrolides, and fluoroquinolones have been found in the genome of *Streptococcus suis* isolated from diseased pigs (32). *S. suis* is an important hemolysin-producing pathogen of swine (33). The decrease in *Streptococcus* abundance may explain the reduced antibiotic resistance and hemolysin levels in the gut microbiome of FBN-fed pigs. Wylensek et al. identified several *Clostridium* species in the cecal microbiota of pigs, such as *C. innocuum*, *C. beijerinckii*, *C. perfringens*, *C. cochlearium*, *C. cadaveris*, *C. porci*, *C. celerecrescens*, and *C. scindens* (31). Among these, *C. porci* was a newly identified species, and a fucosyltransferase-encoding gene was found in its genome (31). *Eubacterium* and *Ruminococcus* are common intestinal genera and have been detected in sows in both prenatal and postnatal periods (34). Yang et al. (35) reported that the abundance of *Eubacterium* and *Ruminococcus* decreased in diarrheic piglets, suggesting that greater numbers of the two genera may indicate a healthier gut microbiota in pigs. A metagenomic analysis showed that *Roseburia inulinivorans* was enriched in the intestinal microbiota of FBN-fed pigs. *R. inulinivorans* is known to produce butyrate from dietary polysaccharides via the secretion of fructofuranosidase, α-amylase, and α-glucanotransferases (36). Function prediction based on the KEGG database indicated that ABC transporter function decreased with FBN supplementation, which is consistent with a previous report showing that traditional Chinese medicine significantly decreased the abundance of ABC transporters in mice (37). The enzymatic activities of gut microbes are very important for nutrient digestion and absorption. Functional insights into the microbiome based on the CAZy database showed that the activities of GT2, GH1, GH13, and GT2 were decreased with FBN treatment. Among these, GT2 was reported to be the most abundant enzyme (family level) in the ileum, cecum, and colon of pigs (16). The decreased activities of these enzyme families may be attributed to the inhibitory effects of some phytochemicals in FBN on microbes capable of producing glycoside transferase and glycoside hydrolase enzymes, which are involved in glycolysis/gluconeogenesis. Cytochrome P450s play an important role in the activation and detoxification of drugs, environmental toxicants, and dietary ingredients. The gut microbiome is involved in the biotransformation of xenobiotics in the host either directly via the production of enzymes that metabolize the xenobiotics or indirectly by influencing host receptors and signaling pathways via metabolite production (38). The CYP51 family is widely distributed in fungi (39). Unexpectedly, FBN supplementation in the diet reduced the abundance of CYP51 in the gut microbiome of the finishing pigs. This may be due to the low abundance of fungi in the gut microbiome of the animals. Additionally, AhR may be associated with the reduced abundance of CYP family proteins given that it acts upstream of CYP genes (27).

## 5. Conclusion

In conclusion, this study was the first to explore the effects of high dietary inclusion levels (20%, *w*/*w*) of *Aspergillus niger*-fermented *Boehmeria nivea* on the composition, structure, and putative functions of the cecal microbiota of finishing pigs. High levels of FBN supplementation in the diet did not affect serum antioxidant capacity, intestinal morphology, or the expression of genes encoding antioxidant-, immune-, and tight junction-related proteins in the liver, except for an observed decrease in *AHR* and *IL6* gene expression levels in the ileum. FBN treatment altered the composition of the gut microbiota at the genus level, as evidenced by the reduced abundance of *Streptococcus* and the enrichment of *Clostridium*, *Eubacterium*, *Ruminococcus*, and *Roseburia inulinivorans*. In addition, the inclusion of FBN in the diet reduced the predicted functions of glycoside hydrolases (GH1, GH13, GH25), which may be associated with pathways involved in glycolysis/gluconeogenesis. Gene functions related to antibiotic resistance and hemolysin-based virulence in the cecal microbiota were attenuated with FBN treatment. These results indicate that high dietary inclusion levels of FBN may not impact gut health in finishing pigs. However, the effects of FBN on growth performance, meat quality, and feed costs require further investigation.

## Data availability statement

The datasets presented in this study can be found in online repositories. The names of the repository/repositories and accession number(s) can be found in the article/Supplementary material.

## Ethics statement

The animal study was approved by all experimental procedures involving animals were approved (2015-8A) by the Animal Care and Use Committee of the Institute of Subtropical Agriculture, Chinese Academy of Science. The study was conducted in accordance with the local legislation and institutional requirements.

## Author contributions

XL: Data curation, Formal analysis, Writing – original draft, Methodology, Software. ZZ: Data curation, Formal analysis, Writing – original draft. FR: Formal analysis, Investigation, Writing – original draft. YJ: Conceptualization, Writing – review & editing, Resources. S-KK: Conceptualization, Writing – review & editing. K-MN: Writing – review & editing, Data curation, Formal analysis, Funding acquisition, Writing – original draft. XW: Writing – review & editing, Funding acquisition, Conceptualization, Project administration.

## Footnotes

^1^ www.majorbio.com

^2^https://github.com/OpenGene/fastp, version 0.20.0

^3^http://bio-bwa.sourceforge.net, version 0.7.9a

^4^ http://metagene.cb.k.u-tokyo.ac.jp/

^5^ http://www.ncbi.nlm.nih.gov/Taxonomy/taxonomyhome.html/index.cgi?chapter=tgencodes#SG1

^6^http://www.bioinformatics.org/cd-hit/, version 4.6.1

^7^http://soap.genomics.org.cn/, version 2.21

^8^ http://www.genome.jp/kegg/

^9^http://www.diamondsearch.org/index.php, version 0.8.35

^10^ http://www.cazy.org/

^11^ https://card.mcmaster.ca/home

^12^ http://www.mgc.ac.cn/VFs/
