# Supplementary material for: Metagenomic characterization of the cecal microbiota community and functions in finishing pigs fed fermented Boehmeria nivea
Source: Front Vet Sci. 2023 Sep 28;10:1253778. doi: 10.3389/fvets.2023.1253778 (PMC10569026; doi:10.3389/fvets.2023.1253778)
Supplement: Supplementary file 1 [file Table_1.docx]

**Supplementary data**

**Table S1. Composition and nutrient levels of the diets (DM basis, %) used in the study.**

| Items | Control group | FBN group (25%, w/w) |
| --- | --- | --- |
| **Ingradients** |  |  |
| Corn | 60.3 | 40.4 |
| Soybean meal | 19.8 | 17.1 |
| Rice bran | 15 | 4.2 |
| Ramie | 0 | 25 |
| Soybean oil | 1.9 | 10 |
| CaHPO_4_ | 1 | 1.1 |
| Limestone | 0.8 | 0.8 |
| Lysine (98%) | 0.2 | 0.4 |
| Threonine (98%) | 0 | 0 |
| Tryptophan (98%) | 0 | 0 |
| Premix ^1)^ | 1 | 1 |
| Total | 100 | 100 |
| **Nutrient levels ^2)^** |  |  |
| Digestive Energy (MJ/kg) | 14.0976 | 14.09607 |
| Crude Protein | 15.3038 | 15.2975 |
| Lysine | 0.94236 | 0.89832 |
| Methionine | 0.25683 | 0.18007 |
| Threonine | 0.58545 | 0.63473 |
| Tryptophan | 0.17 | 0.1276 |
| Ca | 0.5919 | 0.59145 |
| Total P | 0.66507 | 0.45666 |
| Available P | 0.23183 | 0.20996 |

^1)^The premix provided the following per kg of diets: VA 8 000 IU，VD 3 000 IU，VE 33.6 IU，VB_2_ 3.2 mg，VB_12_ 12 μg，nicotinic acid 16 mg，pantothenic acid 10 mg，biotin 0.168 mg，folic acid 1.28 mg，Cu 11.2 mg，Fe 140 mg，Zn 65.6 mg，Mn 37.6 mg，I 1.52 mg，Se 0.30 mg

^2)^Nutrient levels were calculated value.
