# Supplementary material for: Metagenomic characterization of the cecal microbiota community and functions in finishing pigs fed fermented Boehmeria nivea
Source: Front Vet Sci. 2023 Sep 28;10:1253778. doi: 10.3389/fvets.2023.1253778 (PMC10569026; doi:10.3389/fvets.2023.1253778)
Supplement: Supplementary file 2 [file Table_2.docx]

**Supplementary data**

**Table S2. The primer information used in the study.**

| Primer | Forward | Reverse | Product size | References |
| --- | --- | --- | --- | --- |
| AhR | CAACATCCCAAGGGTCCACA | CATGGACACAGCTCCACCAT | 189 | In this study |
| CYP1A1 | TGGGTCTCCTTCTTTACGCC | CCCAACAGAACTGGGCTCAA | 288 | In this study |
| HO-1 | CGCTCCCGAATGAACACTCT | TGGTCCTTAGTGTCCTGGGT | 137 | In this study |
| Nrf2 | TGCAGCTTTTGGCAGAGACA | AGGAGCAATGAAGACTGGGC | 119 | In this study |
| IL-1β | AAAGGGGACTTGAAGAGAG | CTGCTTGAGAGGTGCTGATGT | 286 | Zhao et al., 2019[1] |
| IL-6 | CAAAGCCACCACCCCTAAC | TCGTTCTGTGACTGCAGCTT | 66 | Zhai et al., 2018 |
| IL-8 | TTCTTCTTTATCCCCAAACTGG | CCACATGTCCTCAAGGTAGGA | 63 | Zhai et al., 2018[2] |
| MyD88 | CCCCAGCGATACCCAGTTT | TCCGACGGCACCTCTTTTC | 152 | In this study |
| TLR4 | GCCATCGCTGCTAACATCATC | CTCATACTCAAAGATACACCATCGG | 108 | In this study |
| Occludin | GTGGGACAAGGAACGTATTTATG | TCTCTCCGCATAGTCCGAAA | 115 | In this study |
| Claudin | CCCGTGCCTTGATGGTAAT | AGAAAGATCACTCCCCCAATG | 142 | In this study |
| GAPDH | AGGTCGGAGTGAACGGA | TGGGTGGAATCATACTGG | 145 | In this study |
| ꞵ-actin | CCACGAAACTACCTTCAACTC | TGATCTCCTTCTGCATCCTGT | 131 | In this study |

**Reference**

1. Zhao, L., et al., *Hippophae rhamnoides polysaccharides protect IPEC-J2 cells from LPS-induced inflammation, apoptosis and barrier dysfunction in vitro via inhibiting TLR4/NF-kappa B signaling pathway.* International Journal of Biological Macromolecules, 2020. **155**: p. 1202-1215.

2. Zhai, Z.Y., et al., *Cecropin A Modulates Tight Junction-Related Protein Expression and Enhances the Barrier Function of Porcine Intestinal Epithelial Cells by Suppressing the MEK/ERK Pathway.* International Journal of Molecular Sciences, 2018. **19**(7).
